# 2333 Impact of spoken sentence predictability on cognitive spare capacity in elderly adults with hearing loss

**DOI:** 10.1017/cts.2018.84

**Published:** 2018-11-21

**Authors:** Cynthia R. Hunter, David B. Pisoni, Dakota Collins, Larry E. Humes

**Affiliations:** 1 Indiana University School of Medicine, Bloomington, IN, USA; 2 Speech Research Laboratory, Department of Psychological and Brain Sciences, Indiana University, Bloomington, IN, USA; 3 Audiological Research Laboratory, Department of Speech and Hearing Sciences, Indiana University, Bloomington, IN, USA

## Abstract

OBJECTIVES/SPECIFIC AIMS: Listening effort is needed to understand speech that is degraded by hearing loss and/or a noisy environment. Effortful listening reduces cognitive spare capacity (CSC). Predictive contexts aid speech perception accuracy, but it is not known whether the use of context reduces or preserves CSC. Here, we compare the impact of predictive context and cognitive load on behavioral indices of CSC in elderly, hearing-impaired adults. METHODS/STUDY POPULATION: Elderly, hearing-impaired adults listened in a noisy background to spoken sentences in which sentence-final words were either predictable or not predictable based on the sentence context. Cognitive load was manipulated by asking participants to remember either short or long sequences of visually presented digits. Participants were divided into low or high cognitive capacity groups based on a pretest of working memory. Accuracy and response times were examined for report of both sentence-final words and digit sequences. RESULTS/ANTICIPATED RESULTS: Preliminary results indicate that accuracy and response times for both words and digits were facilitated by sentence predictability, suggesting that the use of predictive sentence context preserves CSC. Response times for both words and digits and accuracy for digits were impaired under cognitive load. Trends were similar across high and low cognitive capacity groups. The preliminary results support the idea that habilitation strategies involving context use could potentially support CSC in elderly, hearing-impaired adults. DISCUSSION/SIGNIFICANCE OF IMPACT: These preliminary results support the concept that habilitation strategies involving context use could potentially support CSC in elderly, hearing-impaired adults.

